# Parental engagement in research on paediatric lower respiratory tract infections in Indonesia

**DOI:** 10.1186/s12887-024-04648-8

**Published:** 2024-03-08

**Authors:** Vincentia Rizke Ciptaningtyas, Tanjung Ayu Sumekar, Quirijn de Mast, Marinus Isaäk de Jonge, Ani Margawati

**Affiliations:** 1 Department of Microbiology, Faculty of Medicine, Universitas Diponegoro, Jl. Prof. H. Soedarto, Semarang, SH, Tembalang, Semarang 50275 Indonesia; 2 Diponegoro National Hospital, Semarang, Indonesia; 3 Department of Laboratory Medicine, Laboratory of Medical Immunology, Radboud Center for Infectious Diseases, Radboud University Medical Center, Nijmegen, the Netherlands; 4 Department of Psychiatry, Faculty of Medicine, Universitas Diponegoro, Semarang, Indonesia; 5 Center for Biomedical Research, Faculty of Medicine, Universitas Diponegoro, Semarang, Indonesia; 6 Department of Internal Medicine, Radboud Center for Infectious Diseases, Radboud University Medical Center, Nijmegen, The Netherlands; 7 Department of Public Health, Faculty of Medicine, Universitas Diponegoro, Semarang, Indonesia

**Keywords:** Lower respiratory tract infections, Parents’ engagement

## Abstract

**Background:**

Lower respiratory tract infections (LRTIs) in children are a major concern in Indonesia as it is the leading cause of morbidity and mortality. Therefore, research on LRTIs is crucial to improve children’s health. However, clinical research in children is challenging due to parental concerns. This study aims to understand parental considerations for taking part in clinical studies on LRTI in the Indonesian context.

**Methods:**

A cross-sectional study using a validated online questionnaire was conducted from November 2021 to March 2022. This study included parents from two public elementary schools and two private primary schools in Semarang, Indonesia. A total of 1236 responses were analysed.

**Results:**

There was a significant association between educational attainment and willingness to participate in general health and LRTI-related research requiring specimen collection; respondents with an advanced educational level were more likely to refuse participation in research. A similar pattern was observed among respondents with smaller families and younger children against participation in LRTI research. Most respondents who indicated not to participate explained that they did not perceive the necessity to take part and expressed their concerns about endangering their child’s health as a consequence of the specimen collection. Most respondents expected a personal benefit and prioritized access to the study results for their child.

**Conclusion:**

Parents’ educational background and family composition are important determinants of parental engagement in research on LRTI in Indonesia. Notably, parents with a lower educational level, having large families, and older children were more inclined to participate. The emphasis on concerns about potential harm and personal benefit underscores the need for a targeted communication strategy.

**Supplementary Information:**

The online version contains supplementary material available at 10.1186/s12887-024-04648-8.

## Background

Lower respiratory tract infections (LRTIs) in children cause a significant burden and result in high morbidity and mortality rates worldwide, as well as in Indonesia [1]. Clinical research is essential to improve our understanding of LRTI in Indonesian children [2]. However, clinical research in children is challenging. Children were considered too vulnerable to be exposed to possible hazards and additional procedures beyond basic care, and were often not included in clinical research studies [3, 4].

The success of such research initiatives depends significantly on the active engagement and informed consent of parents [5, 6]. Throughout the consent or assent procedure, inadequate conveyance of information might lead to misunderstanding by the parents [7–9]. Furthermore, parents are generally uncertain about their children’s participation in clinical research [10].

It was previously shown in other countries that parents who volunteered to let their children take part in medical research had less social support and tended to have stronger health-seeking behaviour [11]. A study on new-borns found that parents’ consent often depends on the physician’s advice because they feel that they have limited knowledge [12]. Another study discovered that parents who declined to participate in research had a relatively higher socioeconomic status, suffered more from decision anxiety, and found it more difficult to decide independently, as compared to consenting parents [13]. The importance of understanding parental engagement is accentuated in the Indonesian context, where healthcare decision-making is influenced by a myriad of social, educational, and economic backgrounds [14–16].

This study investigates the complexity of parental decision-making in the context of paediatric LRTI research in Indonesia involving specimen collection. In contrast to previous studies that were conducted in clinical settings, our study follows a more comprehensive approach in a community setting by studying the future participation of parents with healthy children during the study.

## Methods

### Study design

A cross-sectional study was conducted in Semarang, Central Java, Indonesia, from November 2021 to March 2022.

### Study population

This study included all parents from two public elementary schools and two private primary schools (nursery, kindergarten, and elementary school) with a large number of students in Semarang. Both parents were approached, but only one parent was allowed to submit an answer for each child. Parents who completed questionnaires more than once or those with invalid identifiers (no data found in the school database) were excluded. Participants provided electronic informed consent prior to starting the survey. The research was authorized by the Ethical Committee of the Faculty of Medicine Universitas Diponegoro (No.376/EC/KEPK/FK-UNDIP/X/2021).

### Data collection

The questionnaires were written in Indonesian and consisted of thirty close-ended, four semi-close, and one open-ended questions developed by the investigators to assess parents’ perspectives on research with children.

The first section of the questionnaire collected demographic information about the respondents: age, gender, profession, marital status, education, income/salary, the total number of children, and the sex and age of the children included in the study.

The second section included questions on respondents’ prior research experience: the decision-maker to participate (semi-closed questions), the previous method used, their impression of the research; their expectations of benefit from research (semi-closed questions), their willingness to let their children participate in future general health research, their preferred form of health research, and, if applicable, the reason they declined to participate (semi-closed questions).

The third section of the questionnaires focused on LRTI research: the willingness of parents to participate in research requiring the collection of samples, their preferred method of sample collection, and, if applicable, their reasons for refusing to participate (semi-closed questions) as well as their preferred age of children to participate. In this section, we provided an illustrated information sheet related to the microbiological methods of sample collection, explained in a comprehensive and straightforward manner.

The final component of the surveys included questions about LRTI diagnosis: children’s prior hospitalization for LRTI, their preferred method of sample collection, their consent to invasive treatments if doctors proposed them, and their reasoning (open-ended questions).

A panel of two paediatricians and three pulmonologists were asked to evaluate the questionnaire in terms of its relevance, importance, and clarity. The experts confirmed that all the questions were essential, as shown by a CVR score of 1. In addition, they indicated that all questions were relevant (I-CVI, S-CVI/UA, S-CVI/Ave 1) and provided valuable comments on a few specific items requiring improvement. The requisite modifications were implemented, and preliminary trials of the questionnaire were carried out.

All parents were given access to the online questionnaire through school social media. We decided to distribute the survey online due to the COVID-19 measures that were in effect during the study period. As suggested by the school administrator, Google Forms survey was used because parents were already accustomed to it. Online responses were uploaded automatically in Google Sheets once the parents submitted the questionnaire.

### Statistical analysis

Each response from parents who had more than one child was treated separately based on the children’s names because parents’ reactions and responses to their different children may vary. Categorical variables are presented as a frequency (percentage). Categorical data (willingness to participate in research, willingness for invasive diagnostic procedures) were analysed using the χ2 test, Fisher’s exact test, and McNemar test as appropriate. Logistic regression was applied to evaluate the effect size of the association between variables and determined using Odds Ratio (95% confidence interval (CI)). All tests were conducted using a two-tailed hypothesis, and a *p* value less than 0.05 was considered statistically significant. Variables with *p* < 0.05 or less in the bivariate analysis were selected for multiple logistic regression analysis using the enter procedure. The data were analysed using SPSS® Version 26.0 (IBM Corp. Release 2019. IBM® SPSS® Statistics for Macintosh, Version 26.0. Armonk, NY).

## Results

### Demographic characteristic

**Fig. 1:**
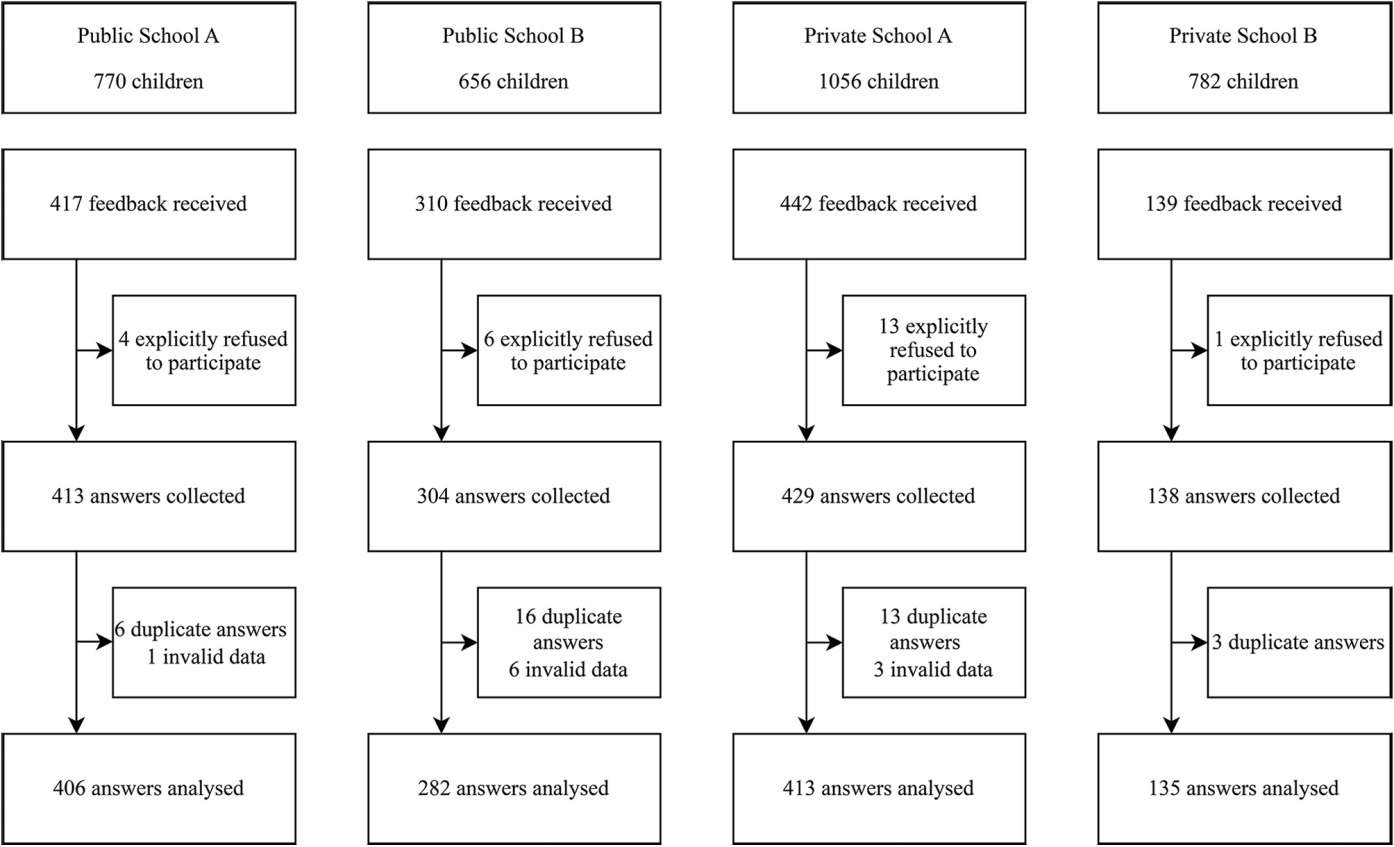
Flow diagram describing the recruitment of participants

All parents from four schools (3264 students) were approached to take part in this study, resulting in 1236 responses (Fig. 1) from parents of 1236 children, 73 of whom had more than one child (data not shown), giving a response rate of 37.9%. Table 1 shows that the majority of the respondents were females, aged between 30 and 39 years, had an advanced educational degree, worked as homemakers, and earned between 2.5 and 5 million Indonesian Rupiah (IDR) per month. The majority of children were male, between the ages of 6 and 12 (elementary school age in Indonesia).

There were statistically significant differences (*p* = 0.002) in the educational level between parents who consented to their children participating in general health research and those who did not (Table 1). Respondents with lower educational levels were less likely to refuse participation in general health research (OR 0.52, 95% CI 0.34 to 0.78, data not shown).

Regarding the willingness to participate in LRTI research, there were statistically significant differences in the gender distribution (*p* = 0.032), educational level (*p* < 0.001), number of children per family (*p* = 0.005), family income (*p* = 0.028), and child age (*p* < 0.001) (Table 1). In this research, respondents with lower educational levels were less likely to refuse participation in LRTI research (OR 0.68, 95% CI 0.52 to 0.89), while respondents with one to two children (OR 1.41, 95% CI 1.10 to 1.80), and respondents with young children (1–5 years) (OR 2.08, 95% CI 1.21 to 3.56) were more often against participating (Supplementary Table 1).

The association between the respondents’ willingness to allow invasive LRTI diagnostic techniques recommended by doctors when their children are unwell and the demographic feature is presented in Table 1, revealing statistically significant differences in educational level (*p* = 0.008), marital status (*p* = 0.018), and family income (< 0.001). Married respondents were less likely to oppose the use of invasive procedures for diagnostic purposes (OR 0.61, 95% CI 0.37 to 0.99), while those with a family income < 2.5 million IDR were more likely to refuse invasive procedures (OR 1.45, 95% CI 1.06 to 2.00) (Supplementary Table 2).

**Table 1:** Characteristics of the study respondents

Variable	Willing to let children participate in general health research		*p*	Willing to let children participate in LRTI research		*p*	Allowing invasive procedures for LRTI diagnosis		*p*	Total (*n* = 1236) N (%)
	No (*n* = 124) N (%)	Yes (*n* = 1112) N (%)		No (*n* = 693) N (%)	Yes (*n* = 543) N (%)		No (*n* = 336) N (%)	Yes (*n* = 900) N (%)		
Parents‡										
Gender			0.690			0.032*			0.509	
Male	23 (18.5)	223 (20.1)		123 (17.7)	123 (22.7)		71 (21.1)	175 (19.4)		246 (19.9)
Female	101 (81.5)	889 (79.9)		570 (82.3)	420 (77.3)		265 (78.9)	725 (80.6)		990 (80.1)
Age			0.751			0.809			0.307	
20–29	6 (4.8)	43 (3.9)		25 (3.6)	24 (4.4)		18 (5.4)	31 (3.4)		49 (4.0)
30–39	64 (51.6)	617 (55.5)		379 (54.7)	302 (55.6)		174 (51.8)	507 (56.3)		681 (55.1)
40–49	50 (40.3)	408 (36.7)		263 (38.0)	195 (35.9)		130 (38.7)	328 (36.4)		458 (37.1)
50–65	4 (3.2)	44 (4.0)		26 (3.8)	22 (4.1)		14 (4.2)	34 (3.8)		48 (3.9)
Educational level (ISCED 11)			0.002*			< 0.001*			0.008*	
Basic + intermediate	33 (26.6)	459 (41.3)		238 (34.3)	254 (46.8)		154 (45.8)	338 (37.6)		492 (39.8)
Advance	91 (73.4)	653 (58.7)		455 (65.7)	289 (53.2)		182 (54.2)	562 (62.4)		744 (60.2)
Marital status			0.557			0.269			0.018*	
Married	115 (92.7)	1046 (94.1)		645 (93.1)	516 (95)		307 (91.4)	854 (94.6)		1161 (93.9)
Widowed	6 (4.8)	35 (3.1)		23 (4.0)	18 (2.4)		19 (5.7)	22 (2.4)		41 (3.3)
Divorced	3 (2.4)	31 (2.8)		20 (2.9)	14 (2.6)		10 (3.0)	24 (2.7)		34 (2.8)
Total number of child/ children in the family			0.149			**0.005**†			0.350	
1	23 (18.5)	184 (16.5)		120 (17.3)	87 (16.0)		65 (19.3)	142 (15.8)		207 (16.7)
2	69 (55.6)	579 (52.1)		386 (55.7)	262 (48.3)		167 (49.7)	481 (53.4)		648 (52.4)
3	26 (21.0)	280 (25.2)		150 (21.6)	156 (28.7)		80 (23.8)	226 (25.1)		306 (24.8)
4	3 (2.4)	59 (5.3)		31 (4.5)	31 (5.7)		21 (6.3)	41 (4.6)		62 (5.0)
5	3 (2.4)	6 (0.5)		6 (0.9)	3 (0.6)		3 (0.9)	6 (0.7)		9 (0.7)
8	0 (0.0)	4 (0.4)		0 (0.0)	4 (0.7)		0 (0.0)	4 (0.4)		4 (0.3)
Occupation			0.067			0.100			0.406	
Laborer	1 (0.8)	38 (3.4)		19 (2.7)	20 (3.7)		15 (4.5)	24 (2.7)		39 (3.2)
Homemakers	50 (40.3)	416 (37.4)		265 (38.2)	201 (37.0)		133 (39.6)	333 (37.0)		466 (37.7)
Merchant	3 (2.4)	31 (2.8)		17 (2.5)	17 (3.1)		8 (2.4)	26 (2.9)		34 (2.8)
Private employees	31 (25.0)	236 (21.2)		155 (22.4)	112 (20.6)		68 (20.2)	199 (22.1)		267 (21.6)
Civil servant	5 (4.0)	58 (5.2)		39 (5.6)	24 (4.4)		16 (4.8)	47 (5.2)		63 (5.1)
Health practitioner	8 (6.5)	62 (5.6)		44 (6.3)	26 (4.8)		24 (7.1)	46 (5.1)		70 (5.7)
Education practitioner	6 (4.8)	35 (3.1)		26 (3.8)	15 (2.8)		9 (2.7)	32 (3.6)		41 (3.3)
Soldier/ police	2 (1.6)	11 (1.0)		6 (0.9)	7 (1.3)		1 (0.3)	12 (1.3)		13 (1.1)
Entrepreneur	12 (9.7)	73 (6.6)		53 (7.6)	32 (5.9)		24 (7.1)	61 (6.8)		85 (6.9)
Other	6 (4.8)	152 (13.7)		69 (10.0)	89 (16.4)		38 (11.3)	120 (13.3)		158 (12.8)
Family income/ month (IDR)			0.386			0.028*			< 0.001*	
< 2,500,000	27 (21.8)	303 (27.2)		162 (23.4)	168 (30.9)		114 (33.9)	216 (24.0)		330 (26.7)
2,500,000–5,000,000	53 (42.7)	418 (37.6)		268 (38.7)	203 (37.4)		125 (37.2)	346 (38.4)		471 (38.1)
5,000,000–10,000,000	31 (25)	230 (20.7)		152 (21.9)	109 (20.1)		60 (17.9)	201 (22.3)		261 (21.1)
10,000,000–20,000,000	8 (6.5)	113 (10.2)		75 (10.8)	46 (8.5)		19 (5.7)	102 (11.3)		121 (9.8)
20,000,000–50,000,000	5 (4.0)	39 (3.5)		31 (4.5)	13 (2.4)		17 (5.1)	27 (3.0)		44 (3.6)
> 50,000,000	0 (0.0)	9 (0.8)		5 (0.7)	4 (0.7)		1 (0.3)	8 (0.9)		9 (0.7)
Children										
Gender			0.233			0.156			0.965	
Male	71 (57.3)	574 (51.6)		374 (54.0)	271 (49.9)		175 (52.1)	470 (52.2)		645 (52.2)
Female	53 (42.7)	538 (48.4)		319 (46.0)	272 (50.1)		161 (47.9)	430 (47.8)		591 (47.8)
Age			0.094			< 0.001*			0.778	
1–5	12 (9.7)	65 (5.8)		58 (8.4)	19 (3.5)		22 (6.5)	55 (6.1)		77 (6.2)
6–12	112 (90.3)	1047 (94.2)		635 (91.6)	524 (96.5)		314 (93.5)	845 (93.9)		1159 (93.8)
History of hospitalization due to LRTI			0.410			0.356			0.440	
No	119 (96.0)	1080 (97.1)		675 (97.4)	524 (96.5)		328 (97.6)	871 (96.8)		1199 (97.0)
Yes	5 (4.0)	32 (2.9)		18 (2.6)	19 (3.5)		8 (2.4)	29 (3.2)		37 (3.0)

ISCED-11: 2011 International Standard Classification of Education

LRTI: Lower Respiratory Tract Infection

*Variables analysed using Pearson’s chi-square test, *p* < 0.05

†Variable analysed using Fisher’s exact test, *p* < 0.05

‡The responses were analysed per included child; 73 parents had more than one child included in this study

### Experience with clinical research

Of all respondents, 159 (12.9%) had their children previously participated in a clinical research study, of whom 80 respondents (50.3%) made an independent decision to enrol their children without first consulting their children, spouse, parents, family, doctor, or school authority (Supplementary Table 3). The most common method of previous research was a survey (42.8%), followed by an observational study in which their children were involved (23.9%), and a combination of a survey and an observational study (20.1%) (Table 2). The majority of respondents with this participation experience rated their experience as good (71.1%), while some rated it as very good (19.5%) or average (9.4%). The advantages (52.8%) and aims (36.5%) of the research were easier to recall than the study methodology (10.0%) or researchers involved (0.6%).

**Table 2:** Previous experience with clinical research and preferences for future clinical research

Type of clinical research	Previous clinical research *n* = 159 (%)	Preferable future clinical research *n* = 1112 (%)
A single type of research		
Observational study	38 (23.9)	156 (14.0)
Survey	68 (42.8)	505 (45.4)
Specimen collection	11 (6.9)	69 (6.2)
Two types of research		
Observational study, survey	32 (20.1)	259 (23.3)
Observational study, specimen collection	1 (0.6)	30 (2.7)
Survey, specimen collection	6 (3.8)	24 (2.2)
Three types of research		
Observational study, survey, specimen collection	3 (1.9)	69 (6.2)

### Personal benefit expectations

A total of 1083 (87.6%) respondents expected a personal benefit. Among them, 809 (74.7%) respondents gave the highest priority to knowing the personal research results for their children above other benefits such as cash, souvenirs, food, free health care services, and general health information (Supplementary Table 4).

### General health research

Out of all the respondent, 1112 (90%) indicated their willingness to become a part of health research in the future (Table 2). When given the option to choose the types of studies (i.e., survey-based, observational, a study including specimen collection, or a combination of these types), 45.4% of the respondents preferred only survey-based studies (Table 2). The main reason for the 124 respondents not willing to participate was the concern that participation would harm their child’s health (Table 3).

**Table 3:** Reasons for parents’ refusal to participate in health research

Reasons not to participate	General health research (*n* = 124*) N (%)	LRTI research (*n* = 693†) N (%)
Concerns about the potential negative effect on the child’s health	58 (46.8)	395 (57.0)
Does not perceive a necessity	46 (37.1)	210 (30.3)
Concerns that the intervention(s) would make the children irritable	31 (25.0)	182 (26.3)
Lack of time	28 (22.6)	63 (9.1)
Concerns about personal data being shared with others	17 (13.7)	67 (9.7)
Lack of personal advantages	7 (5.6)	23 (3.3)
Concerned about being stigmatized, recognized as suffering from a disease	4 (3.2)	27 (3.9)
The child is too young	0 (0.0)	7 (1.0)
The child refused	0 (0.0)	5 (0.7)
Concerned that medical intervention will lead to psychological trauma of the child	0 (0.0)	3 (0.4)
Not sure about the relevance of the study	0 (0.0)	3 (0.4)
Concerns about the COVID-19 pandemic situation	1 (0.8)	5 (0.7)
Unspecified	1 (0.8)	0 (0.0)

*43 parents had more than one reasons

†198 parents had more than one reasons

### LRTI etiology research

A total of 543 (56.1%) respondents were willing to participate in research on LRTI, for which sample collection is needed. There is a clear association between willingness to participate in general health research and willingness to participate in LRTI research. Respondents who were against participation in general health research were also unwilling to let enrol their child in LRTI research (*p* < 0.001, McNemar test). The three most preferable methods of sampling were the non-invasive approaches: urine collection only (23.9%), a combination of expectorating sputum and urine collection (18.8%), and expectorating sputum only (10.5%) (Supplementary Table 5).

The main reasons for not being willing to participate in LRTI research were the same as those who refused to join general health research, i.e., concerns that the study would harm their children (Table 3). When the respondents were asked at what age their children would be permitted to participate in LRTI research, 222 (32.0%) answered at 17 years of age, and 287 (41.4%) answered that they would never allow their children to participate.

All 1236 respondents were asked to choose their preferred microbiological sample strategy for diagnosing LRTI in children (using the same set as the question related to the preferable microbiological sampling procedure for research). Same as for the LRTI research, the majority of the respondents chose the non-invasive method; the three most preferable methods of choice for sampling were urine collection only (19.3%), expectorating sputum only (17.2%), and a combination of expectorating sputum and urine collection (13.7%). The nasopharyngeal swab was preferred by 1.7% of respondents (Supplementary Table 5).

Nine hundred (72.8%) respondents agreed to an invasive diagnostic test procedure for their children if their doctor requested it. There was a significant association between consent to an invasive microbiological test procedure and willingness to participate in LRTI research. Relatively more respondents who agreed to participate in LRTI research also agreed to an invasive diagnostic approach (*p* < 0.001, McNemar test). Those who agreed stated that they did so to determine the exact etiology (32%) for the sake of their children’s health (27.7%) and to demonstrate their trust in doctors (25.7%). In comparison, those who disagreed expressed concern that the procedures could adversely affect their children’s health (44.3%) (Table 4).

**Table 4:** Respondents’ reasons for willingness to allow invasive LRTI procedures

Reasons	N (%)
**Willing to allow (or not against) invasive LRTI-related sample collection procedures (*n*** **= 900)**	
Ascertainment of the correct diagnosis to enable timely and accurate treatment	304 (33.8)
For the benefit of the health of children in general	249 (27.7)
Trust in the physician	231 (25.7)
Feeling compelled	35 (3.9)
Only when the indication is clear	33 (3.7)
Only if the child is willing	3 (0.3)
To gain a better understanding of the underlying causes of disease	2 (0.2)
Unspecified	12 (1.3)
No answer	31 (3.4)
**Against invasive sample collection procedures (*n*** **= 336)**	
Concerns about the potential negative effect on the child’s health	149 (44.3)
Does not understand the necessity for invasive procedures	50 (14.9)
Convinced that easier techniques will also provide the answer	26 (7.7)
The child is too young	18 (5.4)
Concerns that the intervention(s) would make the children irritable	18 (5.4)
The child refused	15 (4.5)
Not sure about the relevance of the study	14 (4.2)
Concerned that the child will suffer from psychological trauma	11 (3.3)
The child had never previously undergone a clinical procedure	5 (1.5)
Lack of trust in the physician	2 (0.6)
Lack of time	1 (0.3)
Unspecified	12 (3.6)
No answer	15 (4.5)

## Discussion

This cross-sectional study was conducted with parents of healthy children in the community setting to investigate factors influencing their decisions to let their children participate in clinical studies primarily focused on LRTI. In this study, we found a significant association between some of the demographic characteristics of our respondents and their willingness to participate in general health and LRTI research.

Relatively more parents with a lower educational level were willing to participate in general health and LRTI research than those with higher levels of education. In a previous study, the influence of the educational level of parents on research participation varies. Studies conducted by Harth et al. [11, 17] showed that parents who allowed their children to take part in clinical studies had low educational attainment, less social support, and displayed more health-seeking behaviour; they were searching for more information and better ways to help their children. Although parents’ level of understanding is generally higher when they have followed higher education, it may be influenced by other factors related to the individual’s values, self-esteem, and personality [17].

Respondents with small families in our study have higher odds of refusing participation in LRTI research. Parents with small families likely have an increased concern for their children following LRTI research that needed clinical sample collection, as shown in previous research. Verbal reports provided by parents have substantiated that parents who are experiencing parenthood for the first time exhibit heightened levels of anxiety about the health of their first children [18]. This phenomenon has also been observed in vaccination research, in which there was a correlation between birth order and elevated levels of parental anxiety [19, 20].

In this study, relatively more respondents with younger children (1–5 years) were less likely to engage in LRTI research. Almost half of the respondents who were against participation stated that they would never allow their children to participate, irrespective of age. This result shows that our respondents considered their children vulnerable and thus took responsibility for making decisions on their behalf. In contrast to this, we also found that some respondents stated that they refused to participate because their children declined. This finding shows that those respondents were considering their children’s consent. Although parents or guardians could override children’s refusal to research participation [21], every researcher should always understand that “the voluntary consent of the human subject is absolutely vital” [22]. In any setting, researchers must consider the child’s role in deciding to participate in research and how that role evolves across the developmental and sociocultural spectrum [23].

More than half of our respondent who previously enrolled their children in clinical studies took their decision independently. Considering the non-invasive nature of the previous studies in which these respondents participated, it is possible that parents felt it easy to make this decision compared to the LRTI-related research as discussed in this study [7]. The benefits derived from research leave a more remarkable impression on respondents who previously participated in health research. A large proportion of the respondents (87.6%) expected a personal benefit, and most prioritized that participation would give them access to research outcomes for their children. This finding is consistent with the previous study showing that direct benefit to children is among the highest priorities [7]. Although research that does not give direct benefit to children participating is not unethical, the researcher may consider giving direct benefit to children over other types of compensation [24].

The main reason for respondents not participating in general health research and LRTI research in particular was related to concerns about the children’s health. These reasons were the same as those who refused an invasive diagnostic approach. The study from Tait et al. [25] shows that parents considered risks an essential aspect before participating in research. Parents are willing to take greater risks in procedures when their child requires treatment [26]. Although the proposed sampling methods in LRTI research and diagnosis were the same, parents preferred the procedures done with doctors in the clinical care setting for diagnostic purposes and not research purposes; they perceived clinical research as an experiment [26, 27]. Parents also thought that the researchers might be inexperienced and not sufficiently skilled for the intervention [28]. Another reason that is thought to be the background for the refusal of respondents to participate in research is the low level of awareness of respondents regarding the benefits of research for the future, and respondents are more focused on more urgent needs. Castillo et al. [29] stated that distrust of medical personnel and lack of awareness regarding the importance of research studies had been identified as factors that influenced research respondents’ participation level.

It is appropriate that we acknowledge the limitations of this study. Although we have constructed our questionnaire with semi-open and open-ended questions, we still cannot fully explore the respondent’s perceptions. Thus, further research is needed to capture the depth and richness of the respondents’ answers through a more comprehensive qualitative method.

## Conclusion

Parents’ educational background and family composition are important determinants of the willingness of Indonesian parents to allow their children to participate in research on lower respiratory tract infections. Notably, parents with a lower educational level, having large families, and older children were more inclined to participate. The emphasis on concerns about potential harm and personal benefit underscores the need for a targeted communication strategy.

### Electronic supplementary material

Below is the link to the electronic supplementary material.

Supplementary Material 1

Supplementary Material 2

Supplementary Material 3

Supplementary Material 4

Supplementary Material 5

## Data Availability

All data generated or analysed during this study are included in this published article and its supplementary information files.
