# Supplementary material for: Parental engagement in research on paediatric lower respiratory tract infections in Indonesia
Source: BMC Pediatr. 2024 Mar 8;24:165. doi: 10.1186/s12887-024-04648-8 (PMC10921691; doi:10.1186/s12887-024-04648-8)
Supplement: Supplementary file 1 — Supplementary Material 1 [file 12887_2024_4648_MOESM1_ESM.docx]

**Supplementary Table 1**. Multivariate logistic regression analysis of association of respondent willingness to participate in LRTI research with dichotomized variables of socio-demographic characteristics of the study respondents

| Covariates | Willing to let children participate in LRTI research | | Crude OR | Adjusted OR (95% CI) | *p* |
| --- | --- | --- | --- | --- | --- |
|  | No  (*n* = 693)  N (%) | Yes  (*n* = 543)  N (%) |  |  |  |
| Parents |  |  |  |  |  |
| Gender |  |  |  |  |  |
| Male | 123 (17.7) | 123 (22.7) | 0.70 | 0.79 (0.59-1.05) | 0.108 |
| Female (Ref) | 570 (82.3) | 420 (77.3) | 1.00 | 1.00 |  |
| Educational level (ISCED 11) |  |  |  |  |  |
| Basic + intermediate | 238 (34.3) | 254 (46.8) | 0.59 | **0.68 (0.52 to 0.89)** | **0.006** |
| Advance (Ref) | 455 (65.7) | 289 (53.2) | 1.00 | 1.00 |  |
| Total number of child/ children in the family |  |  |  |  |  |
| 1-2 | 506 (73.0) | 349 (64.3) | 1.50 | **1.41 (1.10 to 1.80)** | **0.006** |
| >2 (Ref) | 187 (27.0) | 194 (35.7) | 1.00 | 1.00 |  |
| Family income/ month (IDR) |  |  |  |  |  |
| Below the minimum regional wage (2,500,000 IDR) | 162 (23.4) | 168 (30.9) | 0.68 | 0.89 (0.66 to 1.20) | 0.466 |
| Above the minimum regional wage (Ref) | 531 (76.6) | 375 (69.1) | 1.00 | 1.00 |  |
| Children |  |  |  |  |  |
| Age |  |  |  |  |  |
| 1 - 5 | 58 (8.4) | 19 (3.5) | 2.52 | **2.08 (1.21 to 3.56)** | **0.008** |
| 6 – 12 (Ref) | 635 (91.6) | 524 (96.5) | 1.00 | 1.00 |  |

ISCED-11: 2011 International Standard Classification of Education

LRTI: Lower Respiratory Tract Infection

Ref: Reference
