# Supplementary material for: Parental engagement in research on paediatric lower respiratory tract infections in Indonesia
Source: BMC Pediatr. 2024 Mar 8;24:165. doi: 10.1186/s12887-024-04648-8 (PMC10921691; doi:10.1186/s12887-024-04648-8)
Supplement: Supplementary file 2 — Supplementary Material 2 [file 12887_2024_4648_MOESM2_ESM.docx]

**Supplementary Table 2**. Multivariate logistic regression analysis of association of respondent willingness to allowing invasive procedures for LRTI diagnosis with dichotomized variables of socio-demographic characteristics of the study respondents

| Covariates | Allowing invasive procedures for LRTI diagnosis | | Crude OR | Adjusted OR (95% CI) | *p* |
| --- | --- | --- | --- | --- | --- |
|  | No  (*n* = 336)  N (%) | Yes  (*n* = 900)  N (%) |  |  |  |
| Parents |  |  |  |  |  |
| Educational level (ISCED 11) |  |  |  |  |  |
| Basic + intermediate | 154 (45.8) | 338 (37.6) | 1.41 | 1.17 (0.87 to 1.58) | 0.300 |
| Advance (Ref) | 182 (54.2) | 562 (62.4) | 1.00 | 1.00 |  |
| Marital status |  |  |  |  |  |
| Married | 307 | 854 | 0.57 | **0.61 (0.37 to 0.99)** | **0.045** |
| Unmarried (Ref) | 29 | 46 | 1.00 | 1.00 |  |
| Family income/ month (IDR) |  |  |  |  |  |
| Below the minimum regional wage (2,500,000 IDR) | 114 (33.9) | 216 (24.0) | 1.63 | **1.45 (1.06 to 2.00)** | **0.022** |
| Above the minimum regional wage (Ref) | 222 (66.1) | 684 (76.0) | 1.00 | 1.00 |  |

ISCED-11: 2011 International Standard Classification of Education

LRTI: Lower Respiratory Tract Infection

Ref: Reference
