# Supplementary material for: Parental engagement in research on paediatric lower respiratory tract infections in Indonesia
Source: BMC Pediatr. 2024 Mar 8;24:165. doi: 10.1186/s12887-024-04648-8 (PMC10921691; doi:10.1186/s12887-024-04648-8)
Supplement: Supplementary file 3 — Supplementary Material 3 [file 12887_2024_4648_MOESM3_ESM.docx]

**Supplementary Table 3**. Decision maker of past research (*n* = 159)

| Decision maker | Frequency | Percentage (%) |
| --- | --- | --- |
| My kid | 2 | 1.3 |
| My kid + teacher or authority figure at school | 1 | 0.6 |
| My kid + my family | 1 | 0.6 |
| My kid + I | 8 | 5.0 |
| My kid + myself + teacher or authority figure at school | 1 | 0.6 |
| My kid + myself + my spouse | 6 | 3.8 |
| My kid + myself + my spouse + teacher or authority figure at school | 1 | 0.6 |
| My kid + myself + my spouse + my family | 1 | 0.6 |
| My kid + myself + my spouse + my family + doctor | 1 | 0.6 |
| Teacher or authority figure at school | 3 | 1.9 |
| My family | 8 | 5.0 |
| My family + teacher or authority figure at school | 1 | 0.6 |
| My parents | 13 | 8.2 |
| My spouse | 5 | 3.1 |
| My spouse + my family | 1 | 0.6 |
| Myself | 80 | 50.3 |
| Myself + teacher or authority figure at school | 6 | 3.8 |
| Myself + my spouse | 16 | 10.1 |
| Myself + my spouse + teacher or authority figure at school | 3 | 1.9 |
| Myself + my spouse + teacher or authority figure at school + doctor | 1 | 0.6 |
