# Supplementary material for: Parental engagement in research on paediatric lower respiratory tract infections in Indonesia
Source: BMC Pediatr. 2024 Mar 8;24:165. doi: 10.1186/s12887-024-04648-8 (PMC10921691; doi:10.1186/s12887-024-04648-8)
Supplement: Supplementary file 4 — Supplementary Material 4 [file 12887_2024_4648_MOESM4_ESM.docx]

**Supplementary Table 4**. Personal benefit expectations (*n* = 1083)

| Personal benefit expectations | Frequency | Percentage (%) |
| --- | --- | --- |
| Cash/non-cash/vouchers | 52 | 4.8 |
| Souvenir | 3 | 0.3 |
| Extra food | 3 | 0.3 |
| Free health care services | 189 | 17.5 |
| Knowing the personal (direct) results of the research examination | 809 | 74.7 |
| Health care information | 20 | 1.8 |
| To have a role in science | 2 | 0.2 |
| Combination of knowing the results of the examination + free health care services | 2 | 0.2 |
| All of the above | 1 | 0.1 |
| Other (unspecified) | 2 | 0.2 |
