# Supplementary material for: Parental engagement in research on paediatric lower respiratory tract infections in Indonesia
Source: BMC Pediatr. 2024 Mar 8;24:165. doi: 10.1186/s12887-024-04648-8 (PMC10921691; doi:10.1186/s12887-024-04648-8)
Supplement: Supplementary file 5 — Supplementary Material 5 [file 12887_2024_4648_MOESM5_ESM.docx]

| Preferable microbiology sampling technique for LRTI | Research  (*n* = 543)  N (%) | Diagnosis  (*n* = 1236)  N (%) |
| --- | --- | --- |
| **One technique** |  |  |
| Expectoration | 57 (10.5) | 212 (17.2) |
| Urine collection | 130 (23.9) | 239 (19.3) |
| Nasopharyngeal swab | 10 (1.8) | 21 (1.7) |
| Oropharyngeal swab | 5 (0.9) | 6 (0.5) |
| Blood collection | 27 (5.0) | 78 (6.3) |
| Sputum induction | 5 (0.9) | 19 (1.5) |
| Gastric aspiration | 1 (0.2) | 1 (0.1) |
| Tracheal aspiration | 1 (0.2) | 3 (0.2) |
| Bronchoalveolar lavage | 0 (0.0) | 0 (0.0) |
| Pleural fluid collection | 0 (0.0) | 3 (0.2) |
| Lung aspiration | 0 (0.0) | 4 (0.3) |
| **Two techniques** |  |  |
| Expectoration, urine collection | 102 (18.8) | 169 (13.7) |
| Expectoration, nasopharyngeal swab | 3 (0.6) | 7 (0.6) |
| Expectoration, oropharyngeal swab | 0 (0.0) | 3 (0.2) |
| Expectoration, blood collection | 4 (0.7) | 19 (1.5) |
| Expectoration, sputum induction | 0 (0.0) | 7 (0.6) |
| Expectoration, pleural fluid collection | 0 (0.0) | 2 (0.2) |
| Urine collection, nasopharyngeal swab | 5 (0.9) | 7 (0.6) |
| Urine collection, blood collection | 22 (4.1) | 50 (4.0) |
| Urine collection, sputum induction | 1 (0.2) | 3 (0.2) |
| Nasopharyngeal swab, oropharyngeal swab | 4 (0.7) | 3 (0.2) |
| Nasopharyngeal swab, blood collection | 4 (0.7) | 4 (0.3) |
| Nasopharyngeal swab, lung aspiration | 1 (0.2) | 0 (0.0) |
| Oropharyngeal swab, blood collection | 0 (0.0) | 1 (0.1) |
| Blood collection, sputum induction | 1 (0.2) | 3 (0.2) |
| Sputum induction, tracheal aspiration | 0 (0.0) | 1 (0.1) |
| Gastric aspiration, tracheal aspiration | 0 (0.0) | 2 (0.2) |
| Gastric aspiration, bronchoalveolar lavage | 1 (0.2) | 0 (0.0) |
| Tracheal aspiration, lung aspiration | 0 (0.0) | 1 (0.1) |
| **Three techniques** |  |  |
| Expectoration, urine collection, nasopharyngeal swab | 8 (1.5) | 15 (1.2) |
| Expectoration, urine collection, oropharyngeal swab | 0 (0.0) | 1 (0.1) |
| Expectoration, urine collection, blood collection | 32 (5.9) | 84 (6.8) |
| Expectoration, urine collection, sputum induction | 7 (1.3) | 9 (0.7) |
| Expectoration, nasopharyngeal swab, oropharyngeal swab | 1 (0.2) | 3 (0.2) |
| Expectoration, nasopharyngeal swab, blood collection | 1 (0.2) | 6 (0.5) |
| Expectoration, nasopharyngeal swab, tracheal aspiration | 0 (0.0) | 1 (0.1) |
| Expectoration, blood collection, sputum induction | 0 (0.0) | 3 (0.2) |
| Expectoration, blood collection, tracheal aspiration | 0 (0.0) | 1 (0.1) |
| Expectoration, blood collection, pleural fluid collection | 1 (0.2) | 0 (0.0) |
| Expectoration, sputum induction, pleural fluid collection | 1 (0.2) | 0 (0.0) |
| Urine collection, nasopharyngeal swab, oropharyngeal swab | 2 (0.4) | 2 (0.2) |
| Urine collection, nasopharyngeal swab, blood collection | 3 (0.6) | 1 (0.1) |
| Urine collection, oropharyngeal swab, blood collection | 0 (0.0) | 1 (0.1) |
| Urine collection, blood collection, sputum induction | 0 (0.0) | 3 (0.2) |
| Urine collection, blood collection, lung aspiration | 1 (0.2) | 0 (0.0) |
| Nasopharyngeal swab, oropharyngeal swab, blood collection | 0 (0.0) | 2 (0.2) |
| Nasopharyngeal swab, blood collection, pleural fluid collection | 0 (0.0) | 1 (0.1) |
| **Four techniques** |  |  |
| Expectoration, urine collection, nasopharyngeal swab, oropharyngeal swab | 13 (2.4) | 14 (1.1) |
| Expectoration, urine collection, nasopharyngeal swab, blood collection | 5 (0.9) | 18 (1.5) |
| Expectoration, urine collection, nasopharyngeal swab, sputum induction | 1 (0.2) | 4 (0.3) |
| Expectoration, urine collection, blood collection, sputum induction | 0 (0.0) | 2 (0.2) |
| Expectoration, urine collection, blood collection, tracheal aspiration | 0 (0.0) | 1 (0.1) |
| Expectoration, urine collection, blood collection, bronchoalveolar lavage | 1 (0.2) | 0 (0.0) |
| Expectoration, urine collection, oropharyngeal swab, blood collection | 3 (0.6) | 1 (0.1) |
| Expectoration, nasopharyngeal swab, oropharyngeal swab, blood collection | 1 (0.2) | 1 (0.1) |
| Expectoration, nasopharyngeal swab, oropharyngeal swab, sputum induction | 0 (0.0) | 2 (0.2) |
| Expectoration, nasopharyngeal swab, oropharyngeal swab, tracheal aspiration | 0 (0.0) | 1 (0.1) |
| Expectoration, nasopharyngeal swab, oropharyngeal swab, pleural fluid collection | 0 (0.0) | 1 (0.1) |
| Urine collection, nasopharyngeal swab, oropharyngeal swab, blood collection | 5 (0.9) | 4 (0.3) |
| Nasopharyngeal swab, oropharyngeal swab, blood collection, sputum induction | 0 (0.0) | 2 (0.2) |
| Nasopharyngeal swab, oropharyngeal swab, blood collection, bronchoalveolar lavage | 0 (0.0) | 1 (0.1) |
| **Five techniques** |  |  |
| Expectoration, urine collection, nasopharyngeal swab, oropharyngeal swab, blood collection | 17 (3.1) | 34 (2.8) |
| Expectoration, urine collection, nasopharyngeal swab, oropharyngeal swab, sputum induction | 1 (0.2) | 0 (0.0) |
| Expectoration, urine collection, nasopharyngeal swab, blood collection, sputum induction | 0 (0.0) | 1 (0.1) |
| Expectoration, urine collection, oropharyngeal swab, blood collection, sputum induction | 0 (0.0) | 1 (0.1) |
| Expectoration, sputum induction, tracheal aspiration, bronchoalveolar lavage, lung aspiration | 0 (0.0) | 1 (0.1) |
| **Six techniques** |  |  |
| Expectoration, urine collection, nasopharyngeal swab, oropharyngeal swab, blood collection, sputum induction | 4 (0.7) | 13 (1.1) |
| Expectoration, urine collection, nasopharyngeal swab, oropharyngeal swab, blood collection, pleural fluid collection | 0 (0.0) | 1 (0.1) |
| Expectoration, urine collection, oropharyngeal swab, blood collection, sputum induction, pleural fluid collection | 0 (0.0) | 1 (0.1) |
| Expectoration, sputum induction, gastric aspiration, bronchoalveolar lavage, pleural fluid collection, lung aspiration | 0 (0.0) | 1 (0.1) |
| **Seven techniques** |  |  |
| Expectoration, urine collection, nasopharyngeal swab, oropharyngeal swab, blood collection, gastric aspiration, tracheal aspiration | 0 (0.0) | 1 (0.1) |
| **Nine techniques** |  |  |
| Expectoration, nasopharyngeal swab, oropharyngeal swab, sputum induction, gastric aspiration, tracheal aspiration, bronchoalveolar lavage, pleural fluid collection, lung aspiration | 0 (0.0) | 1 (0.1) |
| **All techniques** | 51 (9.4) | 128 (10.4) |
